# New Insights Into the Regulation of Lipoprotein Metabolism by PCSK9: Lessons From Stable Isotope Tracer Studies in Human Subjects

**DOI:** 10.3389/fphys.2021.603910

**Published:** 2021-02-10

**Authors:** Qidi Ying, Dick C. Chan, Gerald F. Watts

**Affiliations:** ^1^School of Medicine, Faculty of Health and Medical Sciences, University of Western Australia, Perth, WA, Australia; ^2^Lipid Disorders Clinic, Department of Cardiology and Internal Medicine, Royal Perth Hospital, Perth, WA, Australia

**Keywords:** *PCSK9*, PCSK9 inhibitor, LDL-cholesterol, lipoprotein(a), lipoprotein metabolism, stable isotope tracer study

## Abstract

Proprotein convertase subtilisin/kexin type 9 (PCSK9) is a convertase enzyme mostly produced by the liver. It is a key regulator of LDL metabolism because of its ability to enhance degradation of the LDL receptor. PCSK9 also regulates the metabolism of lipoprotein(a) [Lp(a)] and triglyceride-rich lipoproteins (TRLs). Its key role in modulating atherosclerotic cardiovascular disease (ASCVD) is supported by genetic studies and clinical outcome trials. Kinetic studies provide mechanistic insight into the role of PCSK9 in regulating the physiology and pathophysiology of plasma lipids and lipoproteins. Kinetic data have demonstrated that plasma PCSK9 concentration is inversely associated with the clearance of LDL in men. Gain-of-function mutations of PCSK9 markedly increase plasma LDL-cholesterol concentrations due to impaired LDL-apoB catabolism. Conversely, PCSK9 deficiency results in low LDL-cholesterol associated with enhanced LDL-apoB clearance. Inhibition of PCSK9 with monoclonal antibodies (such as evolocumab or alirocumab) lowers plasma LDL-cholesterol and apoB levels chiefly by upregulating the catabolism of LDL particles in healthy individuals. As monotherapy, PCSK9 inhibitor reduced Lp(a) concentrations by decreasing the production rate. However, as combination therapy, it reduced the plasma concentration of Lp(a) by increasing the fractional catabolism of Lp(a) particles. In statin-treated patients with high Lp(a), PCSK9 inhibition lowers plasma Lp(a) concentrations by accelerating the catabolism of Lp(a) particles. The effect of PCSK9 inhibition on TRL metabolism has been studied in healthy individuals and in patients with type 2 diabetes. These findings suggest that PCSK9 appears to play a less important role in TRL than LDL metabolism. Kinetic studies of PCSK9 inhibition therapy on lipoprotein metabolism in diverse high risk patient populations (such as familial hypercholesterolemia) and new therapeutic combination also merit further investigation.

## Introduction

Elevated low-density lipoprotein (LDL)-cholesterol is a major cause of atherosclerotic cardiovascular disease (ASCVD) (Mach et al., 2020). The plasma concentration of LDL-cholesterol may be physiologically determined by a combination of the hepatic secretion of triglyceride-rich very-low density lipoprotein (VLDL), peripheral conversion of VLDL to LDL and, to a greater extent, clearance of LDL particles by the liver via the LDL receptor (LDLR) (Havel, 2010). Apolipoprotein B-100 (apoB-100), the major protein component of LDL, is essential for the binding of LDL particles to the LDLR, which is required for cellular uptake and degradation of LDL (Brown and Goldstein, 1975, 1986). Previous evidence suggests that proprotein convertase subtilisin/kexin type 9 (PCSK9) is a key regulator of LDLR involved in the metabolism of apoB-100 containing lipoproteins (Seidah et al., 2003; Park et al., 2004; Horton et al., 2009). PCSK9 inhibition reduces plasma concentrations of apoB-containing lipoproteins, including lipoprotein(a) [Lp(a)] (O’Donoghue et al., 2019; Bittner et al., 2020). Hence, understanding the role of PCSK9 in the homeostasis and therapeutic regulation of lipoprotein metabolism is therefore fundamentally and clinically important.

The primary aim of this review is to summarize recent findings on the role of PCSK9 on lipoprotein kinetics from human studies chiefly carried out *in vivo* with stable isotopically labeled isotopomers. We also review the mechanisms of action of PCSK9 inhibition on lipoprotein metabolism that have contributed to knowledge in this field.

## What Is PCSK9?

Proprotein convertase subtilisin/kexin type 9 is a serine protease expressed predominantly in the liver and the intestine. *In vitro* and animal studies demonstrate that PCSK9 binds to the LDLRs, leading to their degradation (Seidah et al., 2003). Briefly, LDLR is internalized into the hepatocyte and trafficked to a lysosome, where it can be either degraded or recycled to the surface of the hepatocyte. In this pathway, circulating PCSK9 binds to the LDLR on the surface of hepatocytes and prevents the LDLR from recycling, making it more susceptible to enzymatic degradation during endocytosis (Lambert et al., 2012). Overexpression of PCSK9 impairs the function of LDLRs, resulting in a reduced clearance of LDL particles from plasma and an accumulation of LDL in the circulation. PCSK9 also appears to interact with receptors other than the LDLR, including VLDL receptor and LDLR-related protein (LRP), leading to impaired clearance of VLDL (Croyal et al., 2020).

## PCSK9 Gene Variants

Mendelian randomization studies have consistently demonstrated that gene variants (e.g., *PCSK9*, *NPC1L1*, and *HMGCR*) are associated with variations in plasma LDL-cholesterol concentrations (Ference et al., 2015, 2017). The effect of mutations in *PCSK9* on LDL-cholesterol depends on whether the mutation causes a gain or loss of function (Benjannet et al., 2012). Gain-of-function (GOF) mutations of *PCSK9* cause an increased level of PCSK9 in circulation. GOF mutation in PCSK9 D374Y is associated with a higher affinity for LDLRs compared with wild-type PCSK9 mutant (Peterson et al., 2008). Patients with this mutation are known to have increased levels of LDL-cholesterol, increased risk of ASCVD and are less responsive to statins (HMG-CoA reductase inhibitors) (Abifadel et al., 2003). PCSK9 has also been proposed to mediate Lp(a) via the LDLR pathway with data showing a significant increase in plasma Lp(a) concentrations in patients with PCSK9 GOF mutations (Alonso et al., 2016; Tada et al., 2016a,b).

In contrast, loss-of-function (LOF) mutations in *PCSK9* result in low plasma PCSK9 levels and less degradation of LDLRs (Fasano et al., 2007; Kent et al., 2017). Patients with LOF mutations in *PCSK9* have been associated with lower plasma LDL-cholesterol levels and protection against ASCVD (Cohen et al., 2006). Moreover, *PCSK9* R46L LOF mutation is associated with lower levels of Lp(a) and reduced risk of aortic valve stenosis (Langsted et al., 2016). Hence, investigating the effect of PCSK9 mutations on the LDL and Lp(a) kinetics is crucial to understand the complex physiological role of PCSK9 on lipoprotein metabolism.

## Stable Isotopic Tracer Methodologies

Measurements of plasma lipid and apolipoprotein concentration are conventionally employed to characterize disorders of lipoprotein metabolism. However, lipoprotein metabolism is complex and abnormal plasma concentrations can result from alterations in the rates of production and/or catabolism of lipoprotein particles in the circulation (Chan et al., 2006). Tracer studies provide data from which kinetic models can be developed and tested against experimental data (Chan et al., 2004a). This approach has provided better understanding of lipoprotein homeostasis and of the pathogenesis of lipoprotein disorders, as well as the kinetic effects of new lipid-regulating agents, such as PCSK9 inhibitors.

Gas chromatography-mass spectrometry (GCMS) and wider availability of stable isotopically labeled isotopomers has been an increasing use of endogenous labeling of apolipoproteins with amino acid precursor molecules in the investigation of lipoprotein kinetics *in vivo*. One of the most commonly used stable isotope tracers for kinetic studies is deuterated labeled-L-Leucine (D3-leucine). Stable isotopically labeled amino acids (such as D3-leucine) are administered intravenously, as a bolus or primed infusion, with serial blood sampling over several hours/days to study the turnover of apoB-containing lipoproteins. The lipoproteins most widely studied have been VLDL, intermediate density lipoprotein (IDL), LDL-apoB, and Lp(a) particles. Isotopic enrichment of the key apolipoproteins is measured by GCMS. Enrichment data (tracer/tracee ratio) are then analyzed via multicompartmental modeling, from which the fractional turnover and conversion rates of lipoproteins in the circulation are derived. Fractional catabolic (or clearance) rate (pool/day) refers to the fraction of tracee lost from a defined plasma pool per day. From these primary kinetic data, together with the corresponding plasma pool sizes of the lipoproteins, absolute transport rates in the circulation are calculated (Chan et al., 2004b,c). For a more detailed review of the methodology, the reader is directed to our earlier published works (Chan et al., 2004b,c).

## Stable Isotopic Tracer Studies in Human Subjects

### Observational Studies

We review a number of kinetic studies investigating the role of PCSK9 in lipoprotein metabolism in humans including healthy individuals, subjects with GOF and LOF mutations in *PCSK9*, as well as in patients with obesity and elevated Lp(a).

### Healthy Subjects

Using stable isotope tracer technology, we reported that plasma PCSK9 concentration was positively associated with LDL-cholesterol, LDL-apoB concentrations, and inversely with LDL-apoB fractional catabolic rate (FCR) in men with a wide range of body mass index (Chan et al., 2009). The significant association between plasma PCSK9 and LDL-apoB was independent of age, obesity status, homeostasis model assessment (HOMA) score and energy intake. This observation is consistent with the postulated physiological effect of PCSK9 on the LDLR pathway. The inverse correlation between plasma PCSK9 and LDL-apoB FCR has also been confirmed in another study with non-diabetic individuals (Sullivan et al., 2011). In this study, plasma PCSK9 concentration was not associated with LDL-apoB FCR in patients with uncontrolled type 2 diabetes, however. This suggests that poor glycemic control may overwhelm the influence of PCSK9 on LDL-apoB FCR catabolism which merits further investigation.

### Subjects With PCSK9 GOF and LOF Mutations

Patients with GOF mutation for *PCSK9* cause autosomal dominant hypercholesterolemia. Using stable isotope labeling, Ouguerram et al. examined the kinetics of VLDL, IDL, and LDL apoB in 2 hypercholesterolemic subjects carrying GOF S127R mutation in *PCSK9*. Compared with healthy controls, high levels of LDL-cholesterol and apoB were due to decreased LDL-apoB FCR and increased VLDL-, IDL-, and LDL-apoB production rate (PR) (Ouguerram et al., 2004). The impaired clearance of LDL from plasma is probably consequent on decreased LDLR activity. The mechanisms for the impact of *PCSK9* GOF mutation on apoB oversecretion need further investigation. It is possible that cholesterol overload in hepatocytes in familial hypercholesterolemia (FH) and/or the diminished LDLR activity itself may enhance hepatic secretion of apoB, as observed in FH patients with *LDLR* mutation.

In a kinetic study of LOF mutation in *PCSK9*, Cariou et al. found that individuals carrying double mutation *PCSK9* R104C/V114A exhibited low plasma LDL-cholesterol and apoB compared with controls (Cariou et al., 2009). This was primarily attributed to an accelerated catabolism of VLDL, IDL, and LDL, by contrast to the impaired apoB catabolism in patients with the GOF mutation S127R. However, the effect of PCSK9 on apoB production in this GOF mutation is inconsistent, showing either increased or decreased apoB production that merits further investigation.

The above kinetic studies have been extended to investigate the role of PCSK9 on Lp(a) metabolism. Apo(a) kinetics in Lp(a) were investigated in patients with *PCSK9* GOF and LOF mutation. Croyal et al. (2020) found that plasma Lp(a) concentrations were only slightly increased in patients with *PCSK9* GOF mutation compared with healthy controls and those with *PCSK9* LOF mutation. However, the FCRs of Lp(a)-apo(a) were similar in all groups, probably owing to small sample size. Hence, the precise role for PCSK9 in Lp(a) catabolism remains to be investigated. It is also possible that increased plasma concentration of PCSK9 in patients with *PCSK9* GOF mutation may upregulate the expression of apo(a) mRNA and, by implication, the production and assembly of Lp(a) particles (Tada et al., 2016a,b). This speculation remains to be further tested experimentally and verified in a wider range of genetically defined subjects.

### Obese Subjects

Obesity is strongly associated with lipid abnormalities and may account for the increased risk of ASCVD. The role of PCSK9 on the metabolism of TRLs in these patients remains unclear. In a postprandial study of obese individuals, we found that plasma PCSK9 concentration was positively associated with postprandial lipemia, as reflected by TRL-apoB-48 total area-under-curve (AUC) and incremental AUC (Chan et al., 2015). PCSK9 levels were also inversely correlated with the FCR of TRL-apoB-48 independent of age, HOMA score, hepatic lipase or lipoprotein lipase. These findings suggest that the catabolism of TRL–apoB-48 in the postprandial state may be coordinated by PCSK9 in obese individuals. In another kinetic study of 39 obese subjects, plasma PCSK9 concentrations correlated significantly with LDL-cholesterol levels. However, there were no significant associations between plasma PCSK9 levels and the PR or FCR of VLDL-triglycerides (Sullivan et al., 2011). While PCSK9 is a key regulator of LDL metabolism, its role in regulating TRL metabolism appears to be less important. However, this notion requires further investigation employing a larger sample of patients and more precise definition of dyslipidemia.

### Subjects With a Wide Range of Lp(a)

Elevated Lp(a) levels are associated with increased ASCVD risk. Tavori et al. found that plasma PCSK9 concentration was directly associated with Lp(a) concentration in patients with high Lp(a) levels (Tavori et al., 2016). This finding is consistent with experimental observations showing that Lp(a) catabolism is regulated by PCSK9 via the LDLR pathway in human HepG2 cells (Romagnuolo et al., 2015). In healthy individuals with a wide range of Lp(a) concentrations, we found that plasma PCSK9 concentration was associated with the FCR of VLDL-, IDL-, and LDL-apoB. However, there were no significant associations between plasma PCSK9 and the PR or FCR of Lp(a) particles (Watts et al., 2017, 2018). Whether there is a significant association between plasma PCSK9 concentration and Lp(a) kinetics in patients with high Lp(a) merits further investigation.

### Interventional Studies

Proprotein convertase subtilisin/kexin type 9 is now an established target for correcting hypercholesterolemia and reducing ASCVD risk in high-risk patients, such as FH. Inhibition of PCSK9 in combination with statins and/or ezetimibe provides a highly effective approach for lowering LDL-cholesterol concentrations in patients with hypercholesterolemia. Monoclonal antibodies (mAbs) targeting PCSK9, such as evolocumab and alirocumab, have been consistently known to significantly lower plasma LDL-cholesterol and the incidence of ASCVD outcomes (Sabatine et al., 2017; Schwartz et al., 2018). Therapeutically, a PCSK9 mAb binds to the PCSK9 protein in the circulation and inhibits PCSK9 binding to the LDLR. As a consequence, the intracellular recycling of LDLR back to the hepatocyte membrane is increased leading to an accelerated catabolism of apoB-containing lipoprotein particles, mainly VLDL, IDL, and LDL, and thus reductions in plasma apoB and LDL-cholesterol levels. The effect of PCSK9 inhibition on lipoprotein metabolism are discussed below, with specific reference to the mechanisms of action (Table 1).

**TABLE 1:** Effect of PCSK9 inhibition with a monoclonal antibody on plasma concentration, pool size, absolute production and fractional catabolism of LDL-, IDL-, and VLDL-apoB-100, TRL-apoB-48 and Lp(a).

Lipoproteins	Plasma Concentration	Kinetic effects			References
		Pool size	Production	Catabolism	
**ApoB-100**					
LDL	↓↓	↓↓	↓↓	↑↑	Reyes-Soffer et al., 2017; Watts et al., 2017
IDL	↓↓	↓↓	↓	↑↑	
VLDL	↓↓	↓↓	↔	↑	
**TRL-apoB-48**	↔	↔	↔	↔	Chan et al., 2018
**Lp(a)**					
Without Statin	↓↓	↓↓	↓↓	↔	Croyal et al., 2018; Watts et al., 2018, 2020
With Statin	↓↓	↓↓	↔	↑↑	

*↑, mild increase; ↑↑, marked increase; ↓, mild decrease; ↓↓, marked decrease; ↔, no change.*

### Healthy Normolipidemic Subjects

Using stable isotope technique, we undertook a study of the effects of atorvastatin (80 mg daily) and evolocumab (420 mg sc every 2 weeks) over 8 weeks on the kinetics of VLDL, IDL, and LDL in the circulation in normal subjects (Watts et al., 2017). We found that both evolocumab and atorvastatin independently increased the FCRs of VLDL, IDL, and LDL. Evolocumab but not atorvastatin lowered the PRs of IDL and LDL, and this was associated with decreases in the plasma pool sizes of these lipoproteins. These results are generally consistent with another kinetic study showing that alirocumab decreased LDL chiefly by increasing IDL- and LDL-apoB FCRs and decreasing LDL-apoB PR (Reyes-Soffer et al., 2017). The findings that evolocumab and alirocumab elevated the catabolism of LDL concurs with their primary effects in binding and lowering circulating PCSK9 and thus enhancing hepatic LDLR activity. Moreover, the incremental effect of dual therapy with evolocumab and atorvastatin on the catabolism of LDL implies that the primary mechanism of action for the two agents can independently enhance LDLR activity, thereby leading to further reduction of LDL-apoB and LDL-cholesterol within a so-called normocholesterolemic range.

**FIGURE 1:**
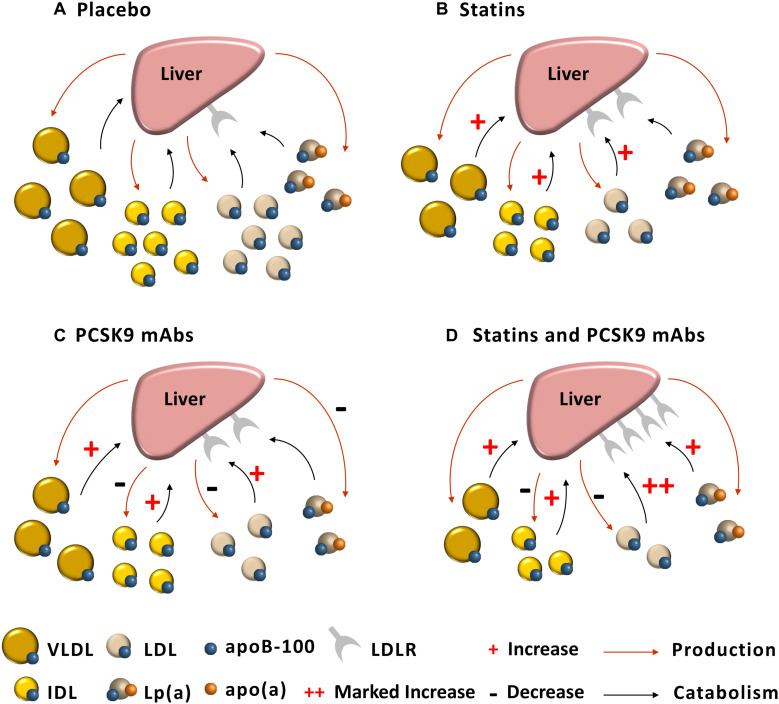
Postulated mechanisms for the effect of statin and PCSK9 mAb on lipoprotein kinetics. **(A)** Placebo: VLDLs, IDLs, and LDLs are preferentially cleared by the LDLR compared with Lp(a). **(B)** Statin (Atorvastatin): LDLR activity is approximately two-fold elevated, resulting in increase in catabolism of VLDLs, IDLs, and LDLs, but no effect on Lp(a) kinetics. **(C)** PCSK9 mAb (Evolocumab): Increased catabolism leads to reduced VLDL concentration; IDL and LDL concentrations decreased due to two-fold elevation in LDLR activity and reduced production of these lipoproteins; PCSK9 mAb reduces Lp(a) concentration by decreasing hepatic production of Lp(a) particles. **(D)** Statin and PCSK9 mAb: VLDL concentration falls due to a doubling in the rate of VLDL catabolism; IDL concentration also falls owing to a doubling in catabolism and reducing in production of IDL; LDL concentration markedly reduced owing to a four-fold elevation in catabolism and halving in the production of LDL; Lp(a) concentration falls due to elevated hepatic clearance of Lp(a) particles (Watts et al., 2017, 2018).

While PCSK9 inhibition and statins enhance hepatic LDLR activity, their effects on Lp(a) kinetics appear to be different. As monotherapy, atorvastatin had no effect on Lp(a) metabolism, whereas evolocumab reduced Lp(a) concentrations by decreasing the production rate. However, as combination therapy, evolocumab reduced the plasma concentration of Lp(a) by increasing the fractional catabolism of Lp(a) particles (Watts et al., 2018). The first mechanistic effect of evolocumab is compatible with a tracer study in non-human primates that alirocumab decreased the production of Lp(a) (Croyal et al., 2018). PCSK9 inhibition may also reduce hepatic production of Lp(a) by decreasing the assembly of Lp(a) particles, which may involve marked decrease in the availability of LDL on the surface of hepatocyte for binding to apo(a) to form Lp(a) particles. It has been reported that, in normal individuals, alirocumab may reduce Lp(a) by increasing the fractional catabolism of Lp(a) particles, but the effect was not statistically significant, reflecting the small sample size used in that study (Reyes-Soffer et al., 2017). The second mechanistic effect of evolocumab may involve supraphysiological upregulation of the activity of LDLRs and decreased competition of Lp(a) with very low concentrations of LDLs for clearance by these receptors (Watts et al., 2018). It remains unclear why in combination with atorvastatin, the effect of evolocumab monotherapy on Lp(a) production is not seen. We have proffered the hypothesis that combination therapy of evolocumab with atorvastatin specifically leads to an increased synthesis of PCSK9 intracellularly that may overcome the primary effect of evolocumab in decreasing the production of Lp(a) particles (Watts et al., 2018). The complexities of this dual mechanism of action for the impact of PCSK9 inhibition on Lp(a) concentration require further investigation (Pirillo and Catapano, 2018) (Figure 1).

### Patients With Elevated Lp(a)

A major challenge in managing patients with elevated Lp(a) is a lack of effective treatment for lowering Lp(a) concentrations. PCSK9 inhibition has been consistently shown to decrease Lp(a) concentrations by up to 30%, with potential reductions in ASCVD events (O’Donoghue et al., 2019). Given that patients with elevated Lp(a) remain at residual risk of ASCVD (Bittner et al., 2020), the mechanisms whereby PCSK9 inhibitors lower Lp(a) concentrations merit investigation. In a recent study of statin-treated patients with evidence of ASCVD and elevated Lp(a), we found that alirocumab significantly decreased plasma Lp(a) concentration chiefly by accelerating the FCR of Lp(a) particles (Watts et al., 2020). As discussed earlier, this effect could be due to potent upregulation of liver receptors and/or less competition between Lp(a) and LDL particles for clearance via these receptors. However, we found that the elevated fractional clearance of Lp(a) with alirocumab could not reduce Lp(a) concentrations into a normal range, implying that, despite PCSK9 inhibition, patients remain at increased residual risk of ASCVD. To address this gap in therapy, treatments that decreasing the synthesis and assembly of Lp(a) will be required to further lower Lp(a) concentration and the attendant risk of ASCVD. This therapeutic target is best achieved using RNA-based therapies, such as apo(a) antisense oligonucleotides and small interfering RNA (Tsimikas et al., 2020).

### Postprandial Studies

Recent work has investigated the impact of inhibiting PCSK9 on TRL metabolism. As discussed earlier, PCSK9 is involved in the intracellular degradation of the VLDL receptor and LRP. Experimental work has shown that PCSK9 deficiency is associated with blunted postprandial hypertriglyceridemia, which is partly related to enhanced hepatic clearance of chylomicrons (Le May et al., 2009). Patients with *PCSK9* LOF mutation have lower levels of fasting and postprandial concentrations of triglycerides and apoB-48 (Ooi et al., 2017). However, it remains unclear whether inhibition of PCSK9 has a potential impact on postprandial TRL metabolism.

### Healthy Subjects

In a study of 10 normal individuals, alirocumab had no significant effect on fasting and postprandial triglyceride levels, nor on apoB-48 responses to a fat load (Reyes-Soffer et al., 2017). However, apoB-48 kinetics were not reported in this study. In a study of 80 healthy individuals, we found that evolocumab significantly decreased the postprandial responses in VLDL-apoB (Chan et al., 2018). However, evolocumab did not change the total nor the incremental responses in plasma triglyceride and apoB-48 (as estimated by AUCs), and also did not have an effect on the kinetics of apoB-48 particles in the postprandial states. However, in the same study, atorvastatin decreased fasting and postprandial apoB-48 concentration by accelerating the catabolism of apoB-48 particles. This mechanistic effect of atorvastatin may relate to reductions in apoC-III and angiopoietin-like 3 concentrations, both of which could enhance the lipolysis and clearance of plasma TRL particles. The above findings suggest that compared with atorvastatin, evolocumab did not accelerate the catabolism of apoB-48 particles because of no effect on lipolysis of nascent chylomicron particles, and less effective hepatic uptake by the VLDL (or LRP) receptors in the postprandial condition.

### Type 2 Diabetic Patients

Postprandial lipidemia may contribute to the increased risk of ASCVD in patients with diabetes. The effect of PCSK9 inhibition on postprandial lipid metabolism has been recently reported in type 2 diabetic patients with dyslipidemia. In a study of 15 diabetic patients on statins, 12 week treatment with evolocumab significantly reduced the postprandial rise in plasma total triglyceride, VLDL_1_-triglyceride, apoC-III, apoB-48 and remnant-like particle (RLP)-cholesterol (Taskinen et al., 2020). The reductions in the postprandial responses were mainly observed in 4 to 6 h in response to the fat load. The authors speculated that evolocumab treatment results in an abundant increase in hepatocyte LDLRs and enhances clearance of remnant particles from the VLDL_1_ and VLDL_2_ density ranges, leading to the decreases observed in the later phase of the postprandial period. Whether reduction in apoC-III with evolocumab contributes to improvement in TRL metabolism merits further investigation.

### Implications and Conclusion

Tracer studies using stable isotopes, mass spectrometry and mathematical modeling methods have played a fundamental role in advancing our knowledge of the homeostasis and pathophysiology of lipid and lipoprotein metabolism. PCSK9 is known to play an integral role in the regulation of LDL metabolism, as supported by recent kinetic studies in healthy individual and in subjects with *PCSK9* GOF and LOF mutations. However, the direct effect of PCSK9 on the metabolism of Lp(a) and TRLs is less convincing and under-researched.

Current guidelines for managing lipid disorders focus on LDL-cholesterol reduction (Mach et al., 2020). The value of LDL-cholesterol lowering for preventing ASCVD events has been re-affirmed by diverse bodies of evidence, including Mendelian randomization studies and clinical trials. While statins are foundational treatment for patients with hypercholesterolemia, a significant proportion of patients, mostly FH patients, do not achieve the recommended LDL-cholesterol concentration target. Inhibition of PCSK9 is now an established approach for reducing residual risk due to elevated LDL-cholesterol in secondary prevention. Recent large outcome trials have consistently demonstrated that PCSK9 mAbs (evolocumab and alirocumab) markedly reduce LDL and improve ASCVD outcomes in high risk patients (with diabetes, metabolic syndrome, chronic kidney disease, peripheral arterial disease, and multiple large artery disease) on statin therapy (Sabatine et al., 2017; Schwartz et al., 2018; Ray et al., 2019; Deedwania et al., 2020). The effectiveness of PCSK9 mAbs in reducing ASCVD events is also found to be most pronounced in patients with high Lp(a) and that the reduction in Lp(a) could also partly mediate the benefit (O’Donoghue et al., 2019; Bittner et al., 2020).

Tracer studies have elucidated the mechanism of action of PCSK9 inhibition for lowering plasma LDL and Lp(a) concentrations, this chiefly entails increase in the catabolism of LDL and Lp(a) particles in high risk patients on background of statin therapy. Its applications will also help to identify new mechanisms by which PCSK9 inhibition modulate TRL and other lipoproteins. These dynamic studies provide unique data for understanding the modes of action and efficacy of lipid regulating therapies in humans and can therefore bear significantly on best clinical practice and the prevention and treatment of ASCVD. The mechanisms of action of other non-antibody approaches to PCSK9 inhibition, including RNA therapeutics, adnectins, and vaccinations, on lipoprotein metabolism remain to be elucidated (Nishikido and Ray, 2018). Kinetic studies of PCSK9 inhibition therapy in diverse high risk patient populations (such as FH) and new therapeutic combinations also merit further investigation.

## Author Contributions

QY developed the conceptual framework and drafted the manuscript. DC and GW suggested topics and discussions, as well as revised the manuscript. All authors approved the manuscript for submission.

## Conflict of Interest

GW has received honoraria for lectures and advisory boards or research grants from Amgen, Arrowhead, AstraZeneca, Esperion, Kowa, Novartis, Regeneron, and Sanofi. The authors declare that the research was conducted in the absence of any commercial or financial relationships that could be construed as a potential conflict of interest.
